# A Real-Time Cup-Detection Method Based on YOLOv3 for Inventory Management

**DOI:** 10.3390/s22186956

**Published:** 2022-09-14

**Authors:** Wen-Sheng Wu, Zhe-Ming Lu

**Affiliations:** School of Aeronautics and Astronautics, Zhejiang University, Hangzhou 310027, China

**Keywords:** inventory management, deep learning, object detection, YOLOv3

## Abstract

Inventory is the basis of business activities; inventory management helps industries keep their inventories stocked with reasonable quantities, which ensures consumers demand while minimizing storage costs. The traditional manual inventory management has low efficiency and a high labor cost. In this paper, we used improved YOLOv3 to detect the cups stored on the warehouse shelves and counted their numbers to realize automated inventory management. The warehouse images are collected by the camera and transmitted to the industrial computer, which runs the YOLOv3 network. There are three feature maps in YOLOv3, the two smaller feature maps and the structure behind them are removed, and the k-means algorithm is used to optimize the default anchor size. Moreover, the detection range is limited to a specified area. Experiments show that, by eliminating those two feature maps, the network parameter is reduced from 235 MB to 212 MB, and detection FPS is improved from 48.15 to 54.88 while mAP is improved from 95.65% to 96.65% on our test dataset. The new anchors obtained by the k-means algorithm further improve the mAP to 96.82%. With those improvements, the average error rate of detection is reduced to 1.61%. Restricted detection areas eliminate irrelevant items to ensure the high accuracy of the detection result. The accurately counted number of cups and its change provide significant data for inventory management.

## 1. Introduction

Inventory is what is stored in the warehouse. More specifically, it includes raw materials, semi-finished products, finished products, spare parts, etc. It is the basis of business activities, but more inventory does not mean more profit. Excessive inventory will occupy a lot of storage space and increase the storage costs. In addition, it will also affect the capital turnover of enterprises, affect the normal arrangement of production plans, and bring greater sales pressure. On the contrary, insufficient inventory will lead to the unmet demand of consumers and the loss of market share of enterprises. One of the objectives of inventory management is to maintain the inventory at a reasonable quantity, such as submitting orders for replenishment in time when the inventory is insufficient. Therefore, it is very important to count the amount of inventory.

The current inventory-counting methods can be divided into three categories including the manual method, the internet of things (IoT) method, and the vision-based method.

Traditionally, industries hire workers to perform the counting job. This is a simple way to realize inventory counting. It does not need any technical conditions and does not have high requirements for workers’ skills or abilities. However, the manual counting method also has many disadvantages. In a large-scale warehouse, the manual counting method is a heavy work; it takes a long time with low efficiency and brings high labor costs. Moreover, Iman et al. [1] indicate that the counting inaccuracy is a significant problem for industries, especially for the retail and manufacturing industries, and it is mainly caused by human error. In order to avoid the shortcomings of manual methods, some new technologies have been used in inventory counting.

With the development of wireless communication technologies and radio frequency identification (RFID), IoT was used in inventory-counting tasks. Tejesh et al. [2] built a warehouse-management system using IoT which recorded inventory quantities and their positions in the warehouse. They first attached a RFID tag with a unique identification number to the product. Then, they used a RFID reader to emit a short-range radio signal to initialize the tag. Products in different stockrooms can be distinguished by different RFID readers used in different stockrooms. After the initialization of the tag, it can be scanned by the RFID reader, and the product information in the tag will be transmitted to the central server with a transmitting node and a receiving node. Finally, all the product information will be shown on a web page through a web server. However, workers are still needed to use the RFID reader. Zhang et al. [3] use a robot to replace the manual labor of operating a RFID scanner. The robot is equipped with an ultrasound sensor, laser scanner, and RGB camera to build a map of the environment. Then, a navigation path is generated to guide the robot. By installing RFID readers on the robot, it can automatically scan inventory and count numbers. However, the RFID method also has some defects, the counting and localization are totally reliant on the RFID tags. In some occasions, the goods or products are small, cheap, and intensive. Using the IoT method, all of them must be attached to RFID tags. It will cost a lot, and some goods or products are not suitable for being adhered to by the tags.

The vision-based method first captures the images of the inventory and then uses the image-processing algorithm to locate and classify the objects to achieve inventory counting. According to different image-processing algorithms used, it can be further divided into a traditional method and a deep learning method.

The Nishchal K. Verma group did a lot of research in this field. In their recent research [4], they propose an inventory-counting method using KAZE features. The algorithm have two input images, one is the object to be detected, and another is the scene image. They extracted KAZE features from those two images and then found KAZE features in the scene image, which correspond to that in object image. Next, by applying the DBSCAN (Density-Based Spatial Clustering of Applications with Noise) clustering algorithm on these features, the bounding box of the object is obtained, and the counting task is finally completed. It performs better than the Fuzzy Histogram, SURF (Speeded Up Robust Features)-based strategy using SVM (Support Vector Machine) and the SURF-based strategy using the DBSCAN clustering method. It can also resist illumination, scaling, rotation, and viewpoint changes. However, the test image contains only four objects at most, the detection performance on dense, tiny objects remains to be tested. The algorithm can only detect one kind of object at once. This is inefficient when dealing with multi-class object detection. In [5], they combine traditional methods with neural networks for inventory detection and counting. The input image is first blurred with a Gaussian filter to reduce noise and then processed by a Sobel edge detector to extract edges. After that, a connected component analysis (CCA) is used to find connected regions and their centroids in the image. Then, each centroid will generate a fixed-size bounding box. Adjacent bounding boxes will be merged to obtain final bounding boxes. Finally, they are sent into a single layer convolutional neural network (CNN) and a softmax layer to obtain their classes. This performs better than Fuzzy Histogram, Cross Correlation, and SURF SVM. However, this method is not end to end and is too complex. More importantly, the results are largely dependent on the Sobel and CCA algorithms, but their performance in complex scenes and small targets needs to be tested.

In the meantime, the deep learning method shows great ability in object detection tasks. Lots of neural network models such as Faster-RCNN (Faster region-based convolutional network) [6], YOLO (You Only Look Once) [7], SSD (Single Shot MultiBox Detector) [8], Retina-Net [9], and YOLOv3 [10] all achieved outstanding results in object-detection tasks and have been widely used in production and life. Wang et al. [11] proposed a method based on SSD called AP-SSD to detect traffic scenes including pedestrians, motor vehicles, and non-motor vehicles, which reached 91.83% mAP on KITTI dataset and 86.36% mAP on Web Dataset. Shi et al. [12] applied YOLOv3 to detecting students’ in class. They used Bayesian optimization and deep separable convolution to improve the YOLOv3 algorithm. It reached 92.2% mAP on Microsoft COCO dataset, which is 1.2% higher than the original YOLOv3. Lawal [13] used YOLOv3 to detect tomatoes and improved it with a label what-you-see approach, densely architecture incorporation, spatial pyramid pooling, and Mish function. It can mostly reach 99.5% AP with a 52 ms detection time.

In our task, there are four classes of cup, and they are all small objects. To achieve real-time cup inventory counting, we tried different methods and found the YOLOv3 deep learning algorithm performed well on our task. To further improve its performance, we made some improvements. We first set a detection area to ignore the cups in the irrelevant area in the input image. Then, we eliminated the two smaller feature maps in YOLOv3 to reduce the network parameters and accelerate the inference process and found it also improves mAP by 1%. Finally, we performed the k-means clustering algorithm on our own dataset to replace YOLOv3’s initial anchors with our new ones, which improved mAP from 96.65% to 96.82%. The proposed method reduced the parameters of the network and achieved high accuracy on real-time small-object inventory counting in complex scenes. It has the potential to be used in other inventory-counting tasks, and the acceleration method can also be used in other similar object-detection scenarios.

## 2. Materials and Methods

### 2.1. YOLOv3 Algorithm

#### 2.1.1. Network Structure

**Figure 1:**
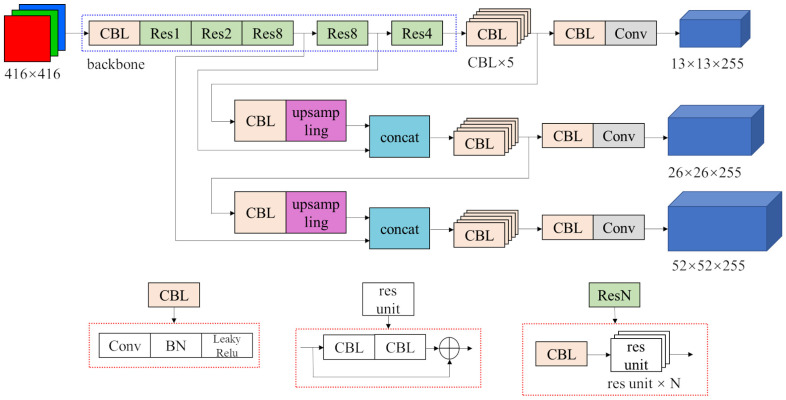
Structure of original YOLOv3 network.

The main method used in our work is the YOLOv3 algorithm. The structure of the original YOLOv3 network is shown in Figure 1.

The input is a RGB three-channel image with a fixed size 416×416. The network structure is mainly composed of two parts. The first part is the part in the blue dotted box. It is the backbone called Darknet-53. However, the last fully connected layer in Darknet-53 is deleted so it actually has 52 convolutional layers. It is responsible for extracting different levels of features from the original image. The rest of the network is a structure called the Feature Pyramid Network (FPN) [14]. Among the features extracted from the backbone, the semantic information of the deep features is more abundant, while the textural information of shallow features is more abundant. The prediction is mainly depended on semantic information, but textural information is also important. What FPN does is combine them together to make the prediction more accurate. The outputs are three tensors that contain the detection results of all the bounding boxes. Their dimensions are 13×13×255, 26×26×255, 52×52×255, and they correspond to 13×13, 26×26, 52×52 feature maps, respectively.

The specific structures of modules such as CBL, res unit, Res1 or Res8, etc., are shown in the red dotted boxes below the network structure. The BN module in CBL represents batch normalization, and leaky Relu is a widely used activation function. The Concat module joins two tensors together in the channel dimension.

#### 2.1.2. Anchor Mechanism

As YOLOv3 has three feature maps, it predicts objects on 3 different scales. The input image is divided into 13×13, 26×26, 52×52 grids.

(1) $$b_{x}=\sigma \left(t_{x}\right)+c_{x}$$

(2) $$b_{y}=\sigma \left(t_{y}\right)+c_{y}$$

(3) $$b_{w}=p_{w}e^{t_{w}}$$

(4) $$b_{h}=p_{h}e^{t_{h}}$$

**Figure 2:**
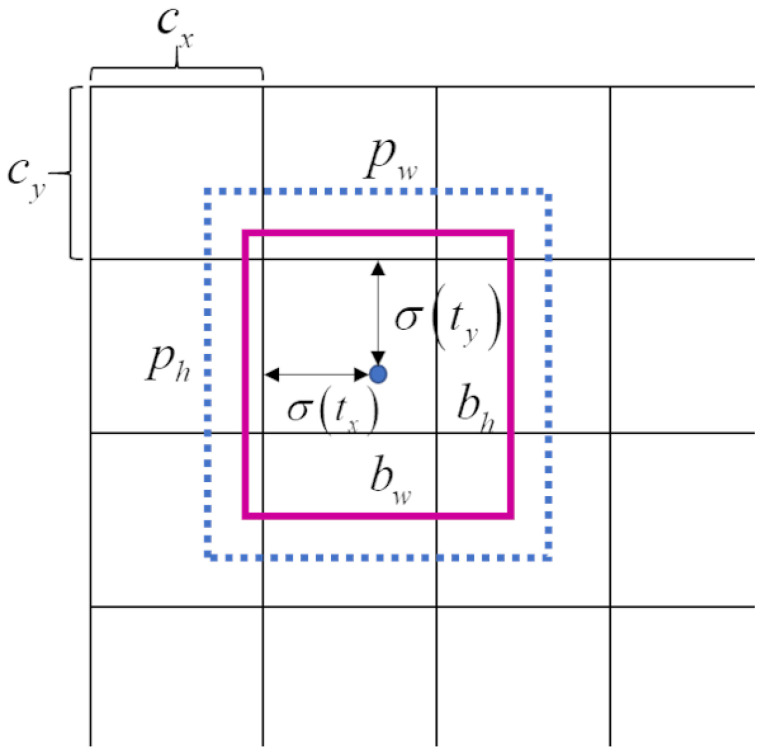
Bounding box coordinates.

Each grid can predict 3 bounding boxes. Each bounding box has 4 coordinates $t_{x}$, $t_{y}$, $t_{w}$, $t_{h}$, which are predicted by the network. Figure 2 and Equations (1)–(4) illustrate the relationship between $t_{x}$, $t_{y}$, $t_{w}$, $t_{h}$ and the width, height, and centroid coordinates of the bounding box. Where $b_{x}$, $b_{y}$ are coordinates of the bounding box centroid, $b_{w}$, $b_{h}$ are the width and height of the bounding box, respectively. $p_{w}$, $p_{h}$ are the prior width and height of the bounding box that is the anchor size. The three feature maps have different receptive fields, so they are responsible for detecting objects of different scales. As a result, the anchor corresponding to each feature map should be different. For each feature map, each grid predicts 3 bounding boxes, so there should be 3 different anchors. Therefore, there are 9 different anchors in total. In original YOLOv3, they are (10×13), (16×30), (33×23), (30×61), (62×45), (59×119), (116×90), (156×198), and (373×326). The corresponding relationship between them and the 3 feature maps is shown in Table 1.

### 2.2. Proposed Improvements

**Figure 3:**
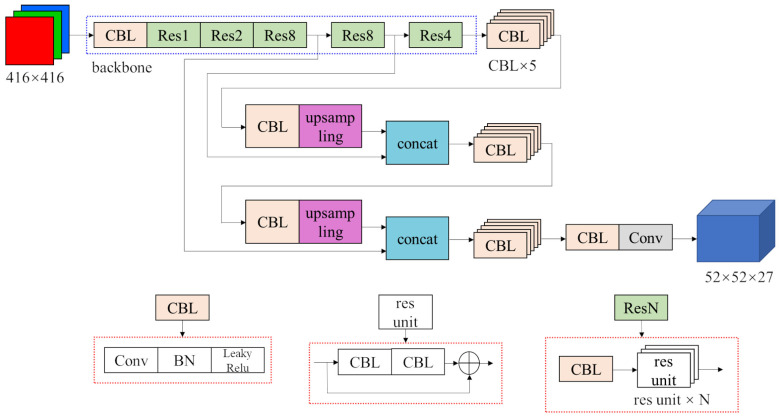
Structure of improved YOLOv3 network.

Among YOLOv3’s 3 feature maps, the two smaller ones have wide receptive fields and are responsible for detecting bigger targets. However, in our task, the targets are all small cups of roughly the same size. Those two feature maps may have little contribution to the detection result’s but they still consume computing resources. Therefore, we eliminate the 13×13 and 26×26 feature maps and the structure behind them to reduce the inference time. The improved structure of the YOLOv3 network is shown in Figure 3. Due to the fact that there are only 4 classes in our detection task, the output tensor of the network has only 27 channels instead of original 255 channels.

Another improvement we made is setting a detection area to ignore the objects beyond that area. The area is rectangular, and we use 4 coordinates to indicate its left, top, right, and bottom edges. Those 4 coordinates are user-defined parameters. After obtaining all the bounding boxes their coordinates will be compared with the area coordinates and only the cups that are totally within the area will be counted. This method is proposed, because the warehouse environment is not always tidy. Some useless cups will be put near the shelf, and they should not be counted.

Finally we redo the k-means clustering algorithm on our own training set to improve the detection accuracy, because there is only one feature map in our network, and only 3 anchors are needed. The smallest 3 original anchors obtained from the Microsoft COCO dataset do not match our cup sizes perfectly. The new anchors we used are shown in Table 2.

### 2.3. Evaluation Methods

In our experiments, we use mAP (mean average precision) and FPS (frame per second) to evaluate the detection results. mAP is used to represent the accuracy of the model, and FPS indicates how many images can be processed by the model in one second, which is the processing speed.

Before defining the mAP, conceptions including IoU, precision rate, and recall rate need to be illustrated. IoU (Intersection over Union) is used to assess the correlation between the ground truth bounding box and the predicted bounding box. It is defined as the following formula:

(5) $$\mathrm{IoU}=\frac{\mathrm{Area}\;\mathrm{of}\;\mathrm{Overlap}}{\mathrm{Area}\;\mathrm{of}\;\mathrm{Union}},$$

where precision rate and recall rate are defined as the following formulas:

(6) $$\mathrm{precision}\;\mathrm{rate}=\frac{\mathrm{TP}}{\mathrm{TP}\;+\;\mathrm{FP}}$$

(7) $$\mathrm{recall}\;\mathrm{rate}=\frac{\mathrm{TP}}{\mathrm{TP}\;+\;\mathrm{FN}}$$

In the above two formulas, TP is the number of true positive objects, while FP and FN are the numbers of false positive objects and false negative objects, respectively. When the IoU between a predicted bounding box and a ground truth is larger than a threshold or equal to the threshold, the predicted bounding box is considered to be a TP. On the other hand, it is a FP. For a ground truth bounding box, if the IoUs of all the predicted bounding boxes are smaller than the threshold, it is considered to be a FN.

The model’s precision rate is different under different recall rates. To better evaluate the model’s performance, the average precision (AP) is defined as following formula, in which *r* stands for the recall rate, and *p(r)* stands for the corresponding precision rate.

(8) $$\mathrm{AP}=\int_{0}^{1}\;p\left(r\right)\;dr$$

mAP is mean AP of all classes of objects. In the following formula, *c* is the number of classes.

(9) $$\mathrm{mAP}=\frac{\sum_{i=0}^{i=c}\;{\mathrm{AP}}_{i}}{c}$$

## 3. Experiment and Analysis

### 3.1. Dataset and Platform

All dataset images are collected from the warehouse by a camera hung on the ceiling. The image size is 1920 pixels × 1080 pixels. The dataset is a mixture of jpg and png images. It is divided into a training set with 378 images, a validation set with 42 images, and a test set with 37 images. Each image contains about 100 different kinds of cups.

**Figure 4:**
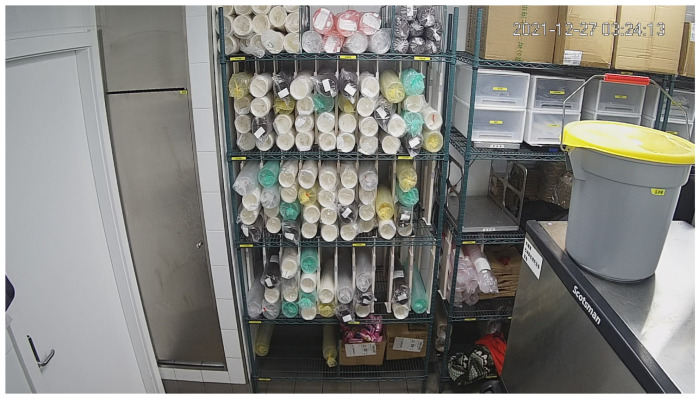
Warehouse scenario in our task.

**Figure 5:**
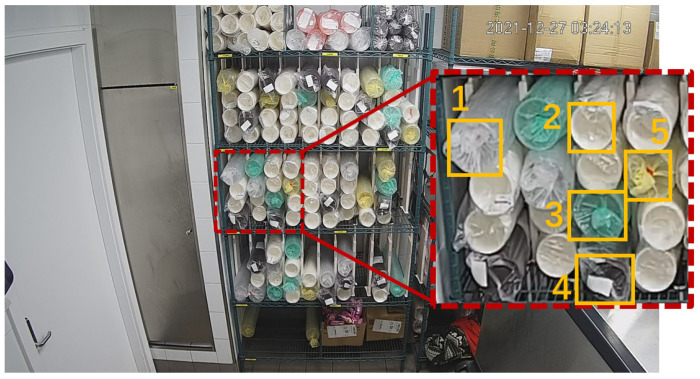
Cup class definition.

Figure 4 above is one of the images in the training set. It is a photo of the warehouse taken by a camera on the roof. In the middle of the picture, there is a shelf on which there are five kinds of cups in total, wrapped in plastic bags of different colors, named as white, green, transparent, black, and yellow, respectively, as shown in Figure 5 and Table 3. A pile of wrapped cups is detected as one object. Under normal circumstances, the shelf and camera are both fixed.

Our task is to detect and count the cups in the middle three layers, while yellow cups are not included in the detected targets according to the project requirements. As a result, cups and some other sundries on the uppermost and the lowermost layers or near the shelf are distractions for our task.

All experiments are carried out on Windows 10 operating system with Inter(R) Core(TM) i7-10700 CPU @ 2.90 GHz, and a Nvidia GeForce GTX 3060 graphics card is used to accelerate the training process.

### 3.2. Features Extracted by Traditional Methods

**Figure 6:**
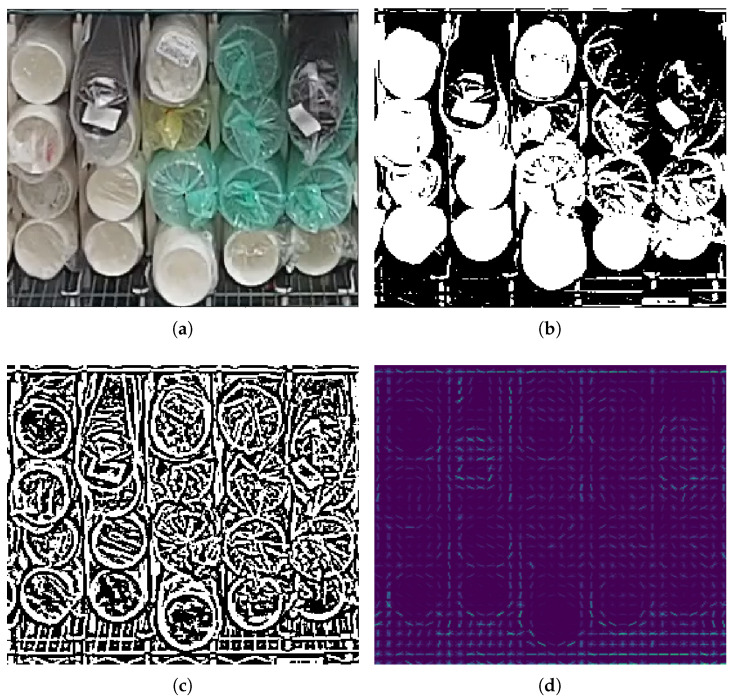
Features extracted by different traditional methods. (**a**) Input image. (**b**) Binarization result with Otsu global threshold. (**c**) Binarization result with adaptive threshold. (**d**) HOG features.

At first, we used traditional algorithms to extract features from the input images. In order to show the features more clearly, we cropped and enlarged a part of the original image, as shown in Figure 6a, which contains 4 classes of cups. In Figure 6b,c show the results of image binarization with the Otsu global threshold [15] and adaptive threshold, respectively. Picture (d) shows the features extracted by the HOG (Histogram of Oriented Gradient) algorithm [16]. Those methods are used a lot in traditional object-detection algorithms, but they have a common characteristic that the color information of input images is lost. There is a label on the black cup, and the wrapping paper of the white cup is less wrinkled. As a result, black and white cups can be distinguished by textural features, but green and yellow cups are almost same in those feature maps. Those feature maps may not be conducive to the classification of the cups. So we turned our attention to the deep learning method.

### 3.3. Detection Result with SSD Algorithm

**Figure 7:**
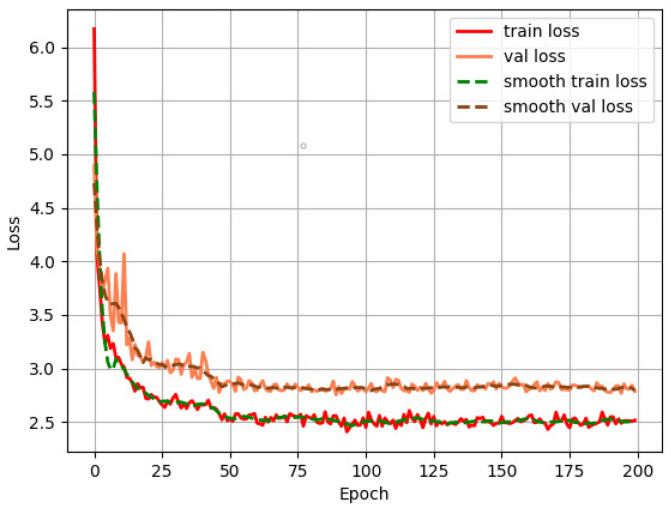
Loss curve of SSD training process.

We first used the SSD algorithm to detect the cups. We chose the VGG (Visual Geometry Group) network as the backbone and set the learning rate as 5 × $10^{-4}$, the batch size as 8, and the input image size as 512 × 512. Figure 7 shows the curve of loss changing with the number of epochs. The loss is high at the beginning and decreases rapidly with the progress of the training. It tends to be stable at epoch 50 and then gradually converges.

**Figure 8:**
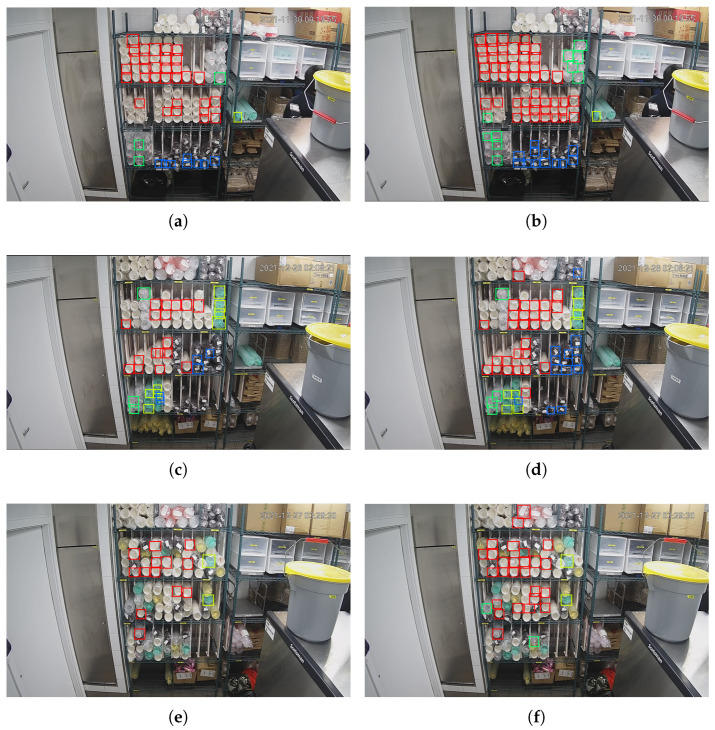
Detection results of SSD algorithm. (**a**,**c**) Detection results under 0.5 confidence threshold and 0.3 NMS IOU. (**b**,**d**) Detection results under 0.3 confidence threshold and 0.1 NMS IOU. (**e**) Detection results under 0.5 confidence threshold and 0.3 NMS IOU. (**f**) Detection results under 0.3 confidence threshold and 0.1 NMS IOU.

To test the performance of the SSD algorithm, we set two sets of parameters on the validation set. We first set the confidence threshold as 0.5 and the NMS (none maximum suppression) IOU threshold as 0.3. The 3 pictures in the left column of Figure 8 show the detecting results. The results show that around half of the cups are not detected, and some of the cups have more than one corresponding predicted bounding box. For the first problem, we guess that is because some predicted bounding boxes do not have high confidence scores, so they are eliminated under the 0.5 confidence threshold. For the second problem, we think the reason is that the NMS threshold is too high, which leads to some predicted bounding boxes having a high IoU. In our task, different cups have almost no overlap area, so the NMS threshold could be very low. According to the above analysis, the confidence threshold was changed to 0.3, and the NMS IOU threshold was changed to 0.1; the detecting results with the same input images are shown in the right column of Figure 8.

After adjusting the confidence threshold and the NMS IOU threshold, the pictures in the right column show that the recall rate is improved, redundant bounding boxes are removed, but false detection situations still exists, and cups beyond the middle 3 layers are also detected.

**Figure 9:**
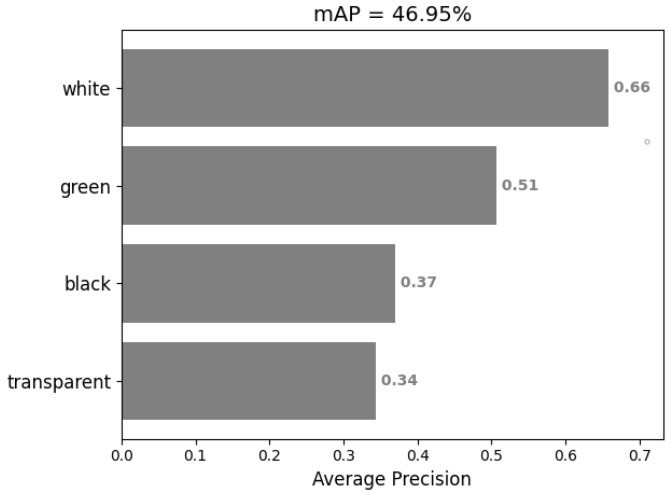
mAP of SSD detection results.

In general, the detection performance has been improved. So, we then ran the algorithm on the test set and used the mAP index to evaluate the detection performance, and the results are shown in the Figure 9. The mAP is 46.95%, which is obviously not high enough to accurately count the numbers of different kinds of cups.

### 3.4. Detection Result with YOLOv3 Algorithm

**Figure 10:**
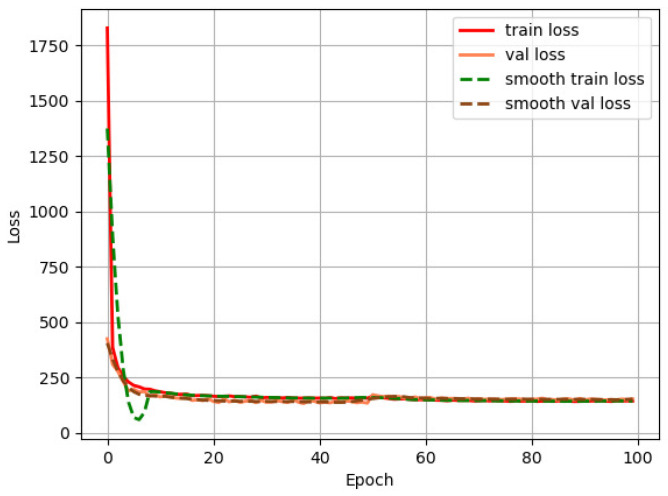
Loss curve of YOLOv3 training process.

We then use the YOLOv3 algorithm to detect the cups. We set the learning rate as $1\times 10^{-4}$, the batch size as 16, and the input image size as 416×416. The loss curves in Figure 10 show that the network quickly converges at around epoch 30.

**Figure 11:**
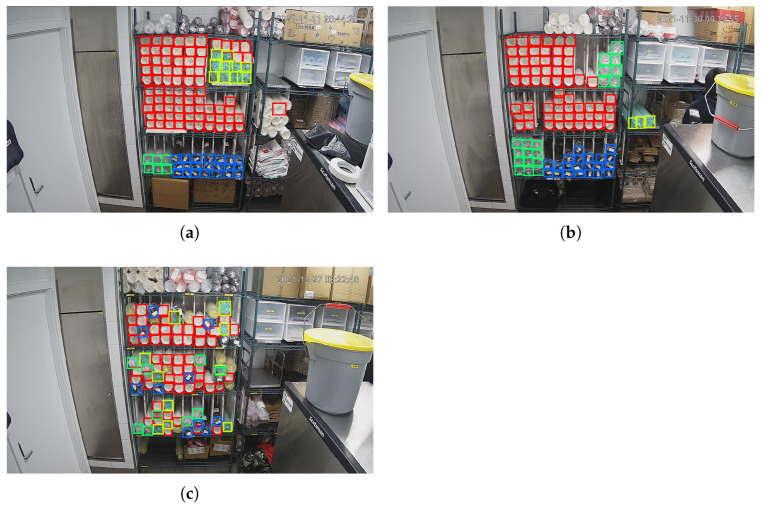
Detection results of YOLOv3 algorithm.

During detection, we set the confidence threshold as 0.6 and the NMS IOU threshold as 0.3. The detection results are shown in Figure 11. Our detection area should be the middle 3 layers of the shelf in the middle of the picture. A white cup in picture (a) and 3 green cups in picture (b) on the right of the shelf are detected and counted, but they are outside the detection area. In picture (c), 4 black cups are missed, and a green cup in the middle layer is wrongly detected as a transparent cup. Table 4 shows the details of the detection results. The error number and error rate are calculated according to the following Equations (10) and (11).

(10) errornumber=networkcountingnumber−errornumber

(11) errorrate=errornumber÷exactnumber×100%

**Figure 12:**
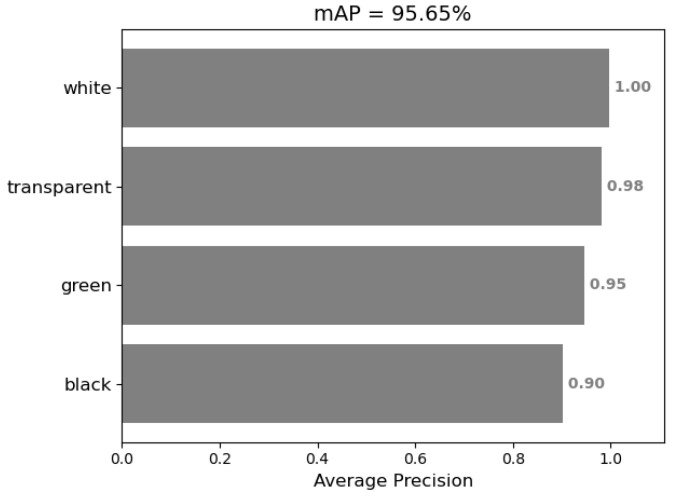
mAP of YOLOv3 detection result.

From Table 4, it can be seen that most of the cups are accurately detected and classified. We further calculated the mAP as shown in Figure 12. The mAP reaches 95.65% which means the detection results are pretty good, and the error is acceptable.

This proves YOLOv3 is capable of completing a cup quantity monitoring task. However, there are still some problems. First, in Figure 11a,b, some cups beyond the detection area are detected, as mentioned before. Second, the anchor sizes are obtained on the COCO dataset by the k-means clustering algorithm; though its performance is good, it may perform better if we redo the clustering step on our own dataset. The average precision (AP) may be further improved, especially for black cups. Finally, the detection FPS (frames per second) is 48.15 on our personal computer with Intel(R) Core(TM) i7-10700 CPU @ 2.90 GHz.However, our algorithm will be run on industrial computer, its computing speed is slower. So, in order to maintain a good real-time performance, the inference process should be optimized.

### 3.5. Detection Result with Improved YOLOv3 Algorithm

#### 3.5.1. Results by Setting Detection Area

**Figure 13:**
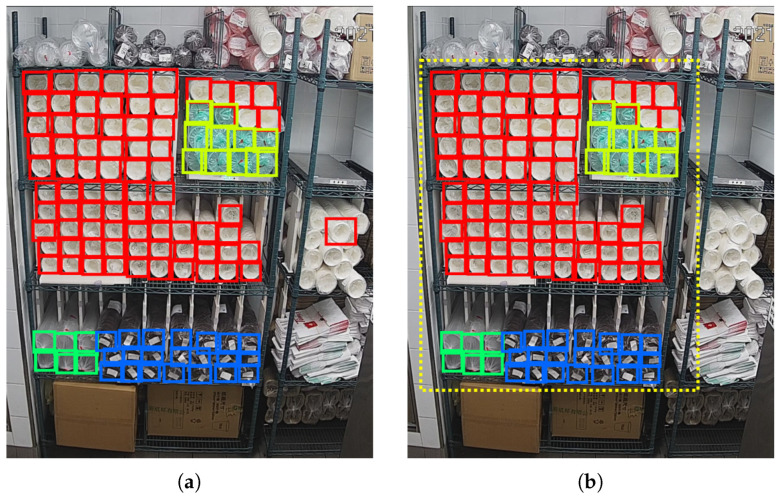

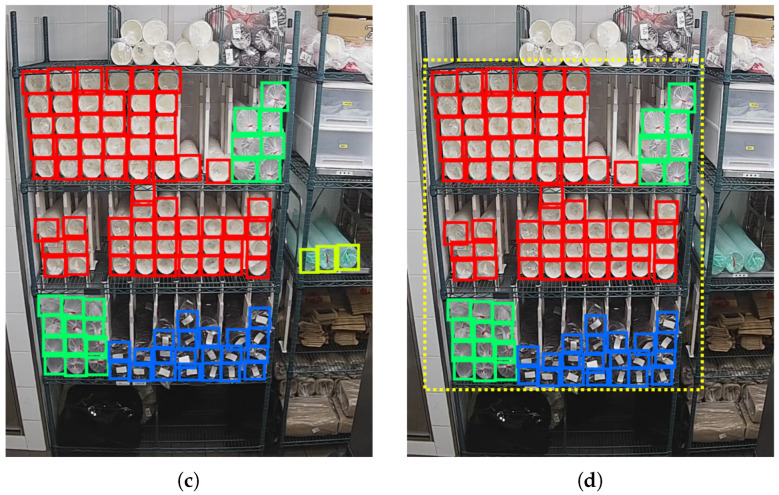
Detection results comparison of setting detection area. (**a**,**c**) Detection results before setting detection area. (**b**,**d**) Detection results after setting detection area.

The first improvement we made is setting a detection area that only contains the middle 3 layers of the shelf, so that the cups beyond the area will be ignored to improve the counting accuracy. The results are shown in Figure 13, in the left column, (a) and (c) are the detecting results before we set the detection area. After setting the detection area, their corresponding results are shown in (b) and (d), respectively. It can be seen that, by setting the detection area, the redundant white cup in (a) and the three redundant green cups in (c) are eliminated. The yellow dashed rectangles in (b) and (d) indicate the detection area we set. They do not appear in the original picture.

**Figure 14:**
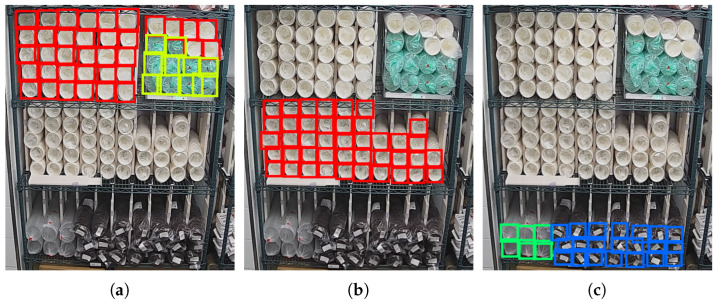
Detection results with different detection area. (**a**) The first layer. (**b**) The second layer. (**c**) The last layer.

Furthermore, the detection area can be adjusted to any rectangular area at any time according to the actual needs. For example, in the three pictures in Figure 14, we set the detection area to detect cups on the first, second, and third layers of the shelf respectively. The detection result of each layer is the same as that in Figure 13b.

#### 3.5.2. Results by Reducing Feature Maps

To reduce the network’s parameters and inference time, we eliminate the 13 × 13 and 26 × 26 feature maps and only retain the 52 × 52 feature map. We use mAP to evaluate the detection accuracy before and after removing the two smaller feature maps and use FPS to evaluate the detection speed. The results are shown in Table 5. Through our improvement, the network’s detection FPS increased from 48.15 to 54.88, and the mAP is improved by 1%.

#### 3.5.3. Results by Resetting Anchors Size

The original anchor sizes in YOLOv3 network are (10,13), (16,30), (33,23), (30,61), (62,45), (59,119), (116,90), (156,198), and (373,326). There are 9 anchor sizes in total, and they correspond to three different feature maps. After removing the two smaller feature maps, we only need 3 anchor sizes. So we set 3 clusters in the k-means algorithm on our training set. The anchor sizes we obtained are (9,15), (10,18), and (11,20), and we retrained the network with the new anchors.

**Figure 15:**
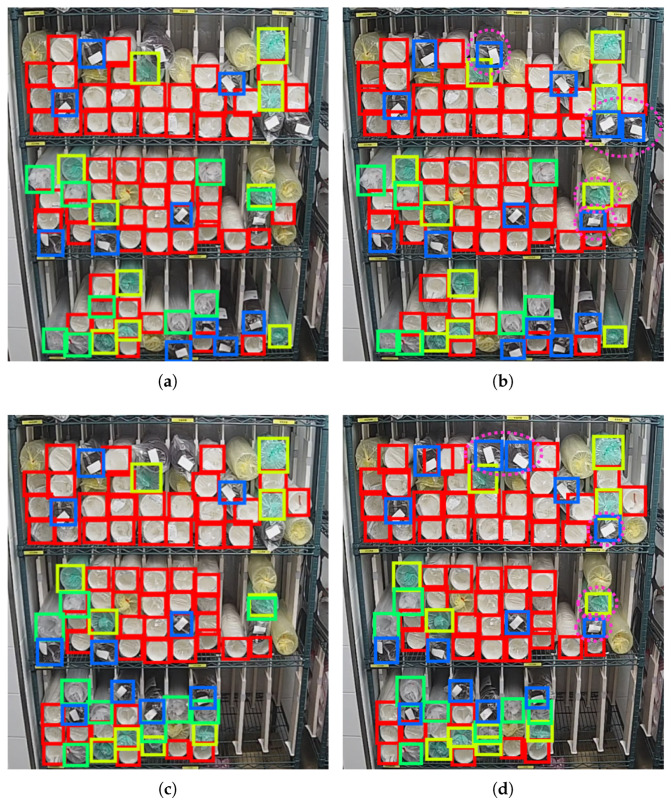
Comparison of detection results of original YOLOv3 and our proposed method. (**a**,**c**) Results of original YOLOv3. (**b**,**d**) Results of our proposed method.

We also trained a YOLOv4 network on the same training set to make a comparison. The following 4 algorithms are run on the same test set, and their confidence thresholds are set as 0.5, while the NMS thresholds are set as 0.3. Table 6 shows the mAP of the detection results. By resetting the anchor sizes, the mAP is increased by 0.17%. It is finally 0.76% higher than YOLOv4. We selected two images in the test set and compared the detection results of the original YOLOv3 and our proposed method, as shown in Figure 15, and the improvements are marked out by fuchsia dotted circles. It can be seen that, under the same conditions, the recall rate of black cups has improved. The false detection of green cups as transparent cups is also eliminated.

To show the detection results more specifically, we counted the total number of different cups in all test set images as the ground truth. We compared it with the cup numbers counted by different algorithms, as shown in Table 7. In both the original YOLOv3 and our improved YOLOv3, we set the confidence threshold as 0.5 and the NMS threshold as 0.3. The error rate is defined in Equation (11), and the average error rate is the average value of the absolute error rate of different kinds of cups. The result shows that our method improves the detection performance on black cups; while the performance on transparent and green cups decrease, in general, it successfully reduces the average error rate from 1.80% to 1.61%, which means the counting is more accurate.

## 4. Discussion

We made some improvements to the YOLOv3 network and applied them to inventory cup detection and counting. The detection mAP reaches 96.82% with 54.88 FPS. This proves that our method can count inventory with high accuracy in real time so that workers can be freed from this work.

The first improvement we made is setting a detection area. Sometimes, we only care about goods or products in specific areas. With this method, the rest of the goods or products can be ignored. At first, we trained another YOLOv3 network to find the shelf and obtain the coordinates of the detection area. However, as mentioned before, the shelf and the camera are both fixed in our task, so the coordinates of the detection area will not change under normal circumstance. So, we set the coordinates as user-defined parameters instead of detecting the shelf, to save time and computing resources. Even in some special cases, when the camera or shelf is moved, the detection area can be adjusted conveniently at any time due to actual needs. User-defined parameters have another advantage that, when the shelf is changed or we are only interested in one of layers of the shelf, the coordinates can be changed easily without retraining a network to obtain the coordinates. However, it is not capable of use in highly dynamic scenes, where training a network to find the detection area is a considerable task. This method can be used in other object-detection tasks as well.

The second improvement we made is eliminating the feature maps and the structures behind them that make no contribution to the final detection results. Through this method, the detection FPS increased from 48.15 to 54.88, while the mAP increased from 95.65% to 96.65%. This is because the contribution of each feature map is different when detecting targets with different scales. In our task, objects are all small cups, so the two smaller feature maps are eliminated. This method can be used in other tiny object-detection tasks and probably in tasks where the objects are approximately the same size to accelerate the detection speed.

The last improvement we made is to refine the anchor size. After performing a clustering algorithm on our own dataset and resetting the anchor size, the mAP increased from 96.65% to 96.82%, which is 0.76% higher than 96.06% of that in YOLOv4. Since the default anchor size of YOLOv3 is obtained on the COCO dataset, this method applies to all tasks which have their own dataset.

The performance of YOLOv4 is better than YOLOv3. However, the model of YOLOv4 is more complex and has more parameters. Considering the limited computing resources of industrial computers and that YOLOv3 is more mature at that time, we chose YOLOv3 as the base of our research and achieved satisfied results. After applying our method on the industrial computer, we found that it may also be capable for YOLOv4. So, in future work, we can apply our improvements on YOLOv4 and examine its performance.

We deployed our algorithm on the industrial computer to process the images collected by the camera in real time and show the results on the web page. It achieves real-time inventory counting with only a 1.61% average error rate. Furthermore, the data can be stored and analyzed to obtain more in-depth inventory-change information; then, corresponding decisions can be made to esnure the normal operation of firms. This builds the foundation of automatic inventory management.

There is only one shelf in our task, and the counting work can be easily completed by a worker. However, compared to manual counting, our method can discover a shortage of inventory in a more timely fashion. Moreover, it has the potential to be used in large-scale warehouses, where it could greatly improve inventory-counting efficiency and reduce labor costs.

## Figures and Tables

**Table 1:** Anchors for different feature maps.

Feature Map Size	Anchor Size
13×13	(116×90), (156×198), (373×326)
26×26	(30×61), (62×45), (59×119)
52×52	(10×13), (16×30), (33×23)

**Table 2:** New anchors obtained on our training set.

Feature Map Size	Anchor Size
52×52	(9×15), (10×18), (11×20)

**Table 3:** Cup class definition.

Box Number	1	2	3	4	5
Class	transparent	white	green	black	yellow

**Table 4:** Counted number and accuracy.

Picture	Cup Class	Network Counting Number	Exact Number	Error Number	Error Rate
	white	79	78	1	1.28%
(a)	green	10	10	0	0
	transparent	6	6	0	0
	black	21	21	0	0
	white	63	63	0	0
(b)	green	3	0	+3	*∞*
	transparent	19	19	0	0
	black	21	21	0	0
	white	53	53	0	0
(c)	green	10	10	0	0
	transparent	9	8	+1	12.50%
	black	10	14	−4	−28.57%

**Table 5:** mAP and FPS comparison before and after reducing feature maps.

	Origin YOLOv3	Optimized YOLOv3
mAP	95.65%	96.65%
FPS	48.15	54.88

**Table 6:** mAP comparison with different improvements and YOLOv4.

	Original YOLOv3	After Eliminating Feature Maps	After Resetting Anchor Size	YOLOv4
mAP	95.65%	96.65%	96.82%	96.06%

**Table 7:** Detection error rate comparison between original YOLOv3 and our method.

	Cup Class	Number	Error Rate	Average Error Rate
**Ground Truth**	white	2052	/	/
	black	859		
	transparent	364		
	green	323		
**Original YOLOv3**	white	2058	0.29%	1.80%
	black	820	−4.54%	
	transparent	367	0.82%	
	green	318	−1.55%	
**Our Method**	white	2058	0.29%	1.61%
	black	820	−0.23%	
	transparent	367	2.20%	
	green	318	−3.72%	

## Data Availability

The study did not report any data.
